# A renewal-equation approach to estimating *R_t_* and infectious disease case counts in the presence of reporting delays

**DOI:** 10.1098/rsta.2024.0357

**Published:** 2025-03-13

**Authors:** Sumali Bajaj, Robin Thompson, Ben Lambert

**Affiliations:** ^1^ Department of Biology, University of Oxford, Oxford, UK; ^2^ Pandemic Sciences Institute, University of Oxford, Oxford, UK; ^3^ Department of Statistics, University of Oxford, Oxford, UK; ^4^ Mathematical Institute, University of Oxford, Oxford, UK

**Keywords:** Bayesian inference, outbreaks, infectious diseases, reproduction number

## Abstract

During infectious disease outbreaks, delays in case reporting mean that the time series of cases is unreliable, particularly for those cases occurring most recently. This means that real-time estimates of the time-varying reproduction number, $R_{t}$, are often made using a time series of cases only up until a time period sufficiently far in the past that there is some confidence in the case counts. This means that the most recent $R_{t}$ estimates are usually out of date, inducing lags in the response of public health authorities. Here, we introduce an $R_{t}$ estimation method, which makes use of the retrospective updates to case time series which happen as more cases that occurred historically enter the health system; these data encode within them information about the reporting delays, which our method also estimates. These estimates, in turn, allow us to estimate the true count of cases occurring most recently allowing up-to-date estimates of $R_{t}$. Our method simultaneously estimates the reporting delays, true historical case counts and $R_{t}$ in a single Bayesian framework, allowing the uncertainty in each of these quantities to be accounted for. We apply our method to both simulated and real outbreak data, which shows that the method substantially improves upon naive estimates of $R_{t}$ which do not account for reporting delays. Our method is available in an open-source fully tested R package, *incidenceinflation*. Our research highlights the value of keeping historical time series of cases since changes to these data can help to characterize nuisance processes, such as reporting delays, which allow these to be accounted for when estimating key epidemic quantities.

This article is part of the theme issue ‘Uncertainty quantification for healthcare and biological systems (Part 1)’.

## 1. Introduction

A range of delays obstructs real-time surveillance efforts during infectious disease outbreaks [1], including the time period from infection to symptom onset, delays in seeking care after symptom onset and reporting mechanisms resulting in variation in case data more reflective of imperfections in the health services than the outbreak signal [2]. Here, we focus on the estimation of the time-varying reproduction number, $R_{t}$, when substantial reporting delays mean the case data are unlikely to be completely recorded until potentially days or weeks after the infections occurred.

Reporting delays typically artificially deflate recent case counts, as those most recently occurring infections are yet to enter health records. This means that naive attempts to estimate the rate of epidemic growth or $R_{t}$ probably understate the seriousness of the current outbreak situation. This issue is well-recognized in outbreak modelling and one approach is to delineate reporting of epidemic quantities according to whether they belong to a so-called *trusted period*, when case data are probably near to complete, and a more recent period when additional extrapolations are necessary to estimate epidemic quantities [3]. Using data on the delay between illness onset and subsequent notification into the health systems, the reporting delay period can be estimated and projections made about recent cases. However, these projections typically require further assumptions to be made, for example, that transmission strength remains the same [3]. Because of these assumptions, such approaches do not allow near real-time estimation of $R_{t}$. An alternative approach is to make full use of the history of incomplete cases stratified by symptom onset data to determine reporting delays and, in turn, estimate recent cases—these approaches have thus far used heuristic statistical models to impute cases [4–9], which do not natively allow the inclusion of knowledge of epidemic spreading rates (e.g. the serial interval distribution) into predictions; it is also not clear that $R_{t}$ estimation is appropriate since the statistical model encodes a different delay distribution from that of the serial interval (which is used to estimate $R_{t}$ from cases [7]). Similar heuristic approaches have also been used to ‘nowcast’ deaths owing to infection in the presence of reporting delays [10]. The trajectories of cases reported with onset on a particular date have also been used to estimate true cases and subsequently to estimate $R_{t}$ [11], although this approach neglects the feedback between the epidemic process and case reporting—i.e. that if $R_{t}$ is higher, we should expect more cases to come to light; it also neglects uncertainty in the delay period.

Our key contribution is to use such data to develop a more mechanistic version of case and $R_{t}$ estimation in the presence of unknown reporting delays. Our method is Bayesian and naturally handles and outputs the joint uncertainty in infections, $R_{t}$ and the reporting delays, facilitating robust real-time decision-making in unfolding outbreaks. We make our method available through a fully tested open-source R package called *incidenceinflation.*^1^

## 2. Method

### (a) Epidemic model

We assume that the count of cases with symptom onset on day t, I(t), follows a renewal process of the form


(2.1) $$\begin{array}{lcr} & I(t)∼f\left(R_{t}\sum_{s=1}^{t-1}\omega_{s}I(t-s)\right), & \end{array}$$

where f(.) denotes either a Poisson or negative binomial probability distribution. Here, $0\leq \omega_{s}\leq 1$ represents an element of a discrete serial interval distribution, which is a probability distribution representing the typical variation in the period from symptom onset of a primary case to symptom onset of cases caused by it. Using a discretized serial interval distribution, equation (2.1), is standard in infectious disease epidemiology, and this implicitly assumes that the serial interval is never negative and that symptomatic cases are caused only by those with symptom onset on previous days; violations to this latter assumption can be handled by using a renewal equation model with time-steps shorter than a day [12].

In this work, we assume that $R_{t}$ is piecewise-constant with the number and length of pieces decided *a priori*; future work could allow these to be estimated from the data [13]. The widths of the $R_{t}$ pieces used in our analyses are shown in electronic supplementary material, table S1.

### (b) Reporting delays

**Figure 1.**
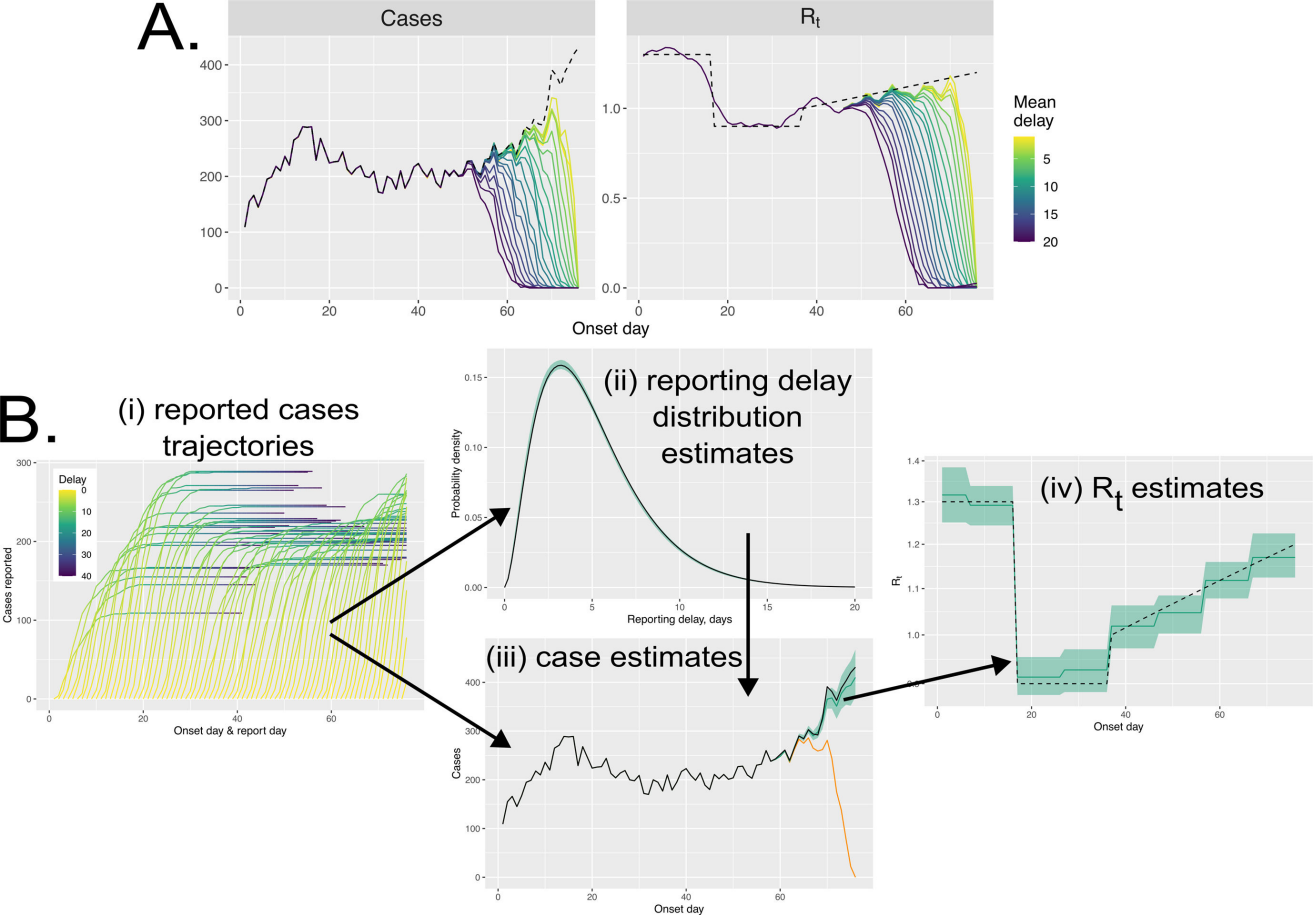
A schematic which shows: (A) how reporting delays affect **$R_{t}$** estimates and (B) our Bayesian approach. In (A), we show how different delay durations cause the cases reported up until the present (coloured lines) to deviate from the true case counts (dashed line); relying on these case counts then results in the most recent $R_{t}$ estimates underestimating the true $R_{t}$ values (dashed lines). In (B), we illustrate our approach, which relies on having access to historical reported case counts for each onset day that can be thought of as *reported case trajectories*; these trajectories contain information both about the delay periods (ii) and the true case counts (iii) and inform our estimates of these; our estimates of case counts (which incorporate uncertainty) are then used to (iv) estimate $R_{t}$, and we assume $R_{t}$ is piecewise-constant.

We suppose that the number of cases thought to have symptom onset on day t, I(t), can be updated as time passes because of a delay in cases entering health systems. Here, we assume that owing to these delays, the number of cases with onset on day t will be less than or equal to the true number of cases onset at that time. This means that, for cases occurring most recently, we tend to underestimate the case counts (figure 1*a*). In reality, retrospective case counts may be revised both upwards and downwards owing to imperfections in reporting which come to light later on; here, we consider only underestimated case counts that arise owing to reporting delays since this case reporting mechanism plagues attempts to obtain contemporary $R_{t}$ estimates.

Our method requires that we have access to historical estimates of case time series (figure 1*b*). We consider the number of cases with symptom onset on day t when observed from some later day, $t^{\prime }>t$, which we represent by $C(t|t^{\prime })$. Since case reporting imperfections are thought to only delay their reception into health systems, we implicitly assume that $C(t|t^{\prime })\leq I(t)$, the true number of cases having onset on day t, and that, as more time passes, the number of cases arising at that time can only increase: if $t^{\prime \prime }>t^{\prime }$ then $C(t|t^{\prime \prime })\geq C(t|t^{\prime })$. The true number of cases with symptom onset on day t, I(t), is then given by the case count at that time when viewed from a time infinitely far into the future: I(t)=C(t|∞). This implicitly means that we assume that, ultimately, all cases are reported—this is unlikely to be true and future work may consider this additional assumption. Although without information regarding the degree of underreporting, it is unclear whether this could be estimated from the case data. If the fraction of cases unobserved remains relatively fixed over time, our method should produce low-bias estimates of $R_{t}$. In reality, this fraction probably varies, particularly at the start of outbreaks, although all predominant methods for $R_{t}$ estimation rely on consistent surveillance.

We assume that the cases detected between day $t^{\prime }$ and later day $t^{\prime \prime }>t^{\prime }$ follow a binomial distribution:


(2.2) $$\begin{array}{lcr} & C(t|t^{\prime \prime })-C(t|t^{\prime })∼\text{binomial}(I(t)-C(t|t^{\prime }),p_{\theta }(t,t^{\prime \prime }-t^{\prime }|t^{\prime })), & \end{array}$$

where the first term on the right-hand side of equation (2.2), $I(t)-C(t|t^{\prime })$, is the number of cases yet to be detected; $p_{\theta }(t,t^{\prime \prime }-t^{\prime }|t^{\prime })$ is the probability that a case arising on day t is observed in the interval $\Delta t=t^{\prime \prime }-t^{\prime }$ given that it has not been observed until day $t^{\prime }$; this probability can be calculated as follows:


(2.3) $$\begin{array}{lcr} & p_{\theta }(t,t^{\prime \prime }-t^{\prime }|t^{\prime })=\frac{S_{\theta }(t|t^{\prime })-S_{\theta }(t|t^{\prime \prime })}{S_{\theta }(t|t^{\prime })}, & \end{array}$$

where $S_{\theta }(t|t^{\prime })$ is the survival function representing the probability that a case occurring on day t has yet to be detected by day $t^{\prime }>t$. This survival probability depends on the parameters, θ, of the distribution characterizing the typical reporting delays: $S_{\theta }(t|t^{\prime }):=1-{\text{CDF}}_{\theta }(t^{\prime }-t)$, where CDF is the cumulative distribution function. In our current software implementation and for generating all results in this article, we assume that the reporting delay distribution is characterized by a gamma distribution with a mean, standard deviation parameterization.

Of course, it may be possible that the true number of cases arising on day t may have already been detected at some later day $t^{\prime }$ meaning that there are no cases remaining at large. We effectively modify equation (2.2) so that detection of cases such that the total exceeds the true number is assigned probability zero.

In real epidemics, the reporting delay probably varies through time and, in our package, we allow the parameters, θ(t), to be piecewise-constant, with the user specifying the number and width of pieces *a priori*. We assume that the day of symptom onset determines the reporting delay distribution.

### (c) Two inference algorithms

We develop a Bayesian method for simultaneously estimating $R_{t}$, the true case counts I(t) and the probability distribution representing the reporting delays, which is characterized by parameters, θ. We have developed two distinct estimation approaches, each of which uses Markov chain Monte Carlo (MCMC) sampling [14,15] to estimate these quantities.

The first of these we term the *Poisson* model since it requires that the renewal distribution (f(.) in equation (2.1)) is a Poisson distribution and makes other restrictive assumptions. The second approach, which we call the *unrestricted* model, allows either a Poisson or negative binomial renewal model, and a wider variety of prior choices. This approach is generally more expensive, though, and requires a more refined initialization to ensure efficient sampling, so we find utility in using both methods.

Both approaches are from the same family of Gibbs-type algorithms. In §2d, we describe the Poisson model; in §2e, we describe the unrestricted model.

### (d) Poisson model

This approach follows a Gibbs-type sampling algorithm comprising the following steps:

1. Generate an estimate ${\hat{R}}_{t}$ conditional on estimates $\{\hat{I}(\tau ){\}}_{\tau =1}^{T}$ where *T* is the last day on which observations occur;
2. Generate estimates, $\{\hat{I}(\tau ){\}}_{\tau =1}^{t}$, conditional on ${\hat{R}}_{t}$ from the previous step and $\hat{\theta }$;
3. Generate an estimate $\hat{\theta }$ conditional on estimates of $\{\hat{I}(\tau ){\}}_{\tau =1}^{t}$ from the previous step.

Like all MCMC methods, the algorithm requires initialization for these three types of quantity and, like for all MCMC methods, this initialization is usually from an arbitrary distribution, ideally one that emulates the posterior distribution. We found inference for the Poisson model insensitive to these starting points and, in all examples we present here, we assumed that $R_{t}=1$ for all time periods; that the initial case histories were given by those reported in the most recent time period; and that (all) reporting periods were characterized by a gamma distribution with a mean of 10 days and standard deviation of 3 days.

#### (i) Sampling $R_{t}$

We assume that $R_{t}$ is piecewise-constant with pieces of time-width $t_{p}$. Within a given piece, i, the $R_{i[t]}$ estimate is then informed by all the cases which were generated using it: $\{I(\tau ){\}}_{\tau =t-t_{p}+1}^{t}$; since the cases within each piece also depend on the history of cases; however, it also depends on all cases before the piece.

If the renewal model given by equation (2.1) follows a Poisson distribution, we can independently sample an $R_{t}$ value within a piece if we know the case history, $\{I(\tau ){\}}_{\tau =1}^{t}$, and assume priors of the form: $R_{i[t]}∼\text{gamma}(a,b)$, representing a prior mean $E[R_{i[t]}]=a/b$. In this situation, we can analytically calculate the posterior distribution of $R_{t}$, which is given by


(2.4) $$\begin{array}{lcr} & R_{i[t]}|\{I(t-s+1){\}}_{s=1}^{t_{p}}∼\text{gamma}\left(a+\sum_{s=1}^{t_{p}}I(t-s+1),b+\sum_{s=1}^{t_{p}}\Lambda (t-s+1)\right), & \end{array}$$

where $\Lambda (t):=\sum_{s=1}^{t-1}\omega_{s}I(t-s)$. In each step of the algorithm, we draw all $R_{i[t]}$ estimates from their conditional posterior distributions of the form given by equation (2.4).

#### (ii) Sampling the true case counts

We now derive the conditional posterior for the true cases $\{I(\tau ){\}}_{\tau =1}^{T}$, which we then independently draw a value from in a Gibbs-type update step. To do so, we write out the numerator of Bayes’ rule considering a single unknown case count, I(t), dropping any terms that do not include I(t). Here, we assume a discrete uniform prior for I(t) up until a maximum of $I(t{)}_{\text{max}}$ which we describe later:


(2.5) $$\begin{array}{r} p(I(t)|I(\text{not }t),\{C(t|t_{j}){\}}_{j=1}^{J},\theta )\propto \left\{ \begin{array}{ll} q(I(t)), & \text{if }I(t{)}_{\text{max}}\geq I(t)\geq C(t|t_{J}),\\ 0, & \text{otherwise}, \end{array}\right. \end{array}$$

where J represents the number of observations made of the case counts arising on a particular day, and


(2.6) $$\begin{array}{rl} q(I(t))= & \left[\prod_{j=1}^{J}\text{\{binomial\}}(C(t|t_{j})-C(t|t_{j-1})|I(t)-C(t|t_{j-1}),p_{\theta }(t,t_{j}-t_{j-1}|t_{j-1}))\right]\times \\ & \text{\{Poisson\}}\left(I(t)|R_{i[t]}\sum_{s=1}^{t-1}\omega_{s}I(t-s)\right)\times \\ & \left[\prod_{\tau =t+1}^{T}\text{\{Poisson\}}\left(I(\tau )|R_{\tau [i]}\sum_{s=1}^{\tau -1}\omega_{s}I(\tau -s)\right)\right], \end{array}$$

where dist(x|ϕ) indicates the probability mass function for the distribution *dist* evaluated at x for parameters ϕ; I(not t) indicates all true case counts except that for cases arising at time t. In equation (2.6), we show the three distinct contributions to the probability: that from the retrospective observations of the cases arising at time t when viewed at later times $t_{j}>t$; the next two terms are associated with the evolution of the underlying state: the first of these is the probability of obtaining I(t) cases at time t given a past history of cases; the second of these concerns case counts *after* t, which depend on I(t) since it forms part of the case history.

The posterior defined by equation (2.6) is non-standard. But because I(t) is a discrete random variable, we can calculate the right-hand side of equation (2.6) across a finite range of integer values of $I(t{)}_{\text{max}}\geq I(t)\geq C(t|t_{J})$, where $t_{J}$ is the latest observation time for retrospective cases; this implicitly means our prior for I(t) given by the middle term on the right-hand side of equation (2.6) is truncated. Then, because we have only a finite set of unnormalized probabilities, we can determine their sum and form a (normalized) discrete probability distribution from which we can independently draw ([15], chapter 14). As long as the uppermost value of I(t) selected is high enough that the probability $p(I(t)>I(t{)}_{\text{max}}|I(\text{not }t),\{C(t|t_{j}){\}}_{j=1}^{J},\theta )<\epsilon$, where ϵ>0 is tiny, then this should not introduce substantial bias (i.e. underestimation) into I(t) estimates. In our current implementation, the user selects a maximum possible count of true cases, $I_{\text{max}}\gg \text{max}(C(t|t^{\prime })),\;\forall t,t^{\prime }$, although future work could refine this to allow a dynamic maximum that ensures $p(I(t)>I(t{)}_{\text{max}}|I(\text{not }t)<\epsilon$, improving the efficiency of the sampling.

#### (iii) Sampling the parameters characterizing the reporting delays

We can again calculate the conditional posterior distribution up to a normalization constant:


(2.7) $$\begin{array}{rl} p(\theta |\{I(\tau ){\}}_{\tau =1}^{T},\{C(t|t_{j}){\}}_{j=1}^{J})\propto p(\theta )\prod_{t=1}^{T}\prod_{j=1}^{J}\text{binomial}( & C(t|t_{j})-C(t|t_{j-1})|I(t)-C(t|t_{j-1}),\\ & p_{\theta }(t,t_{j}-t_{j-1}|t_{j-1})), \end{array}$$

where p(θ) is the prior on θ. In our current implementation of the method, we model the delays as being generated from a gamma distribution with parameters, (μ,σ), representing the mean delay (μ) and standard deviation in delay (σ). In our current software, each of these parameters are assigned independent gamma priors.

In each iteration, we use the random walk Metropolis algorithm ([15], chapter 13) to draw values of θ from the posterior defined by equation (2.7).

### (e) Unrestricted model

The unrestricted model is also a Gibbs-type algorithm but comprises two steps:

1. Generate estimates: ${\hat{R}}_{t}$, $\hat{\theta }$ (and if a negative binomial model is used, an estimate of the overdispersion parameter, $\hat{\kappa }$) conditional on estimates of $\{\hat{I}(\tau ){\}}_{\tau =1}^{T}$ from the previous step;
2. Generate estimates, $\{\hat{I}(\tau ){\}}_{\tau =1}^{T}$, conditional on ${\hat{R}}_{t}$ from the previous step and $\hat{\theta }$.

Step 2 of this algorithm is the same as step 2 described in §2d(ii), except we allow users to optionally replace the Poisson distribution in equation (2.6) by a negative binomial distribution; this introduces an overdispersion parameter, κ>0, where, as κ→∞, the distribution approaches a Poisson.

To allow for more general models of the process, we use Stan’s NUTS sampler in step 1 [16]. In all examples, we used 10 Stan iterations each time step 1 was called, and our updated parameter estimates were those output in the final of these iterations. Stan’s NUTS algorithm uses various adaptive phases where reasonable hyperparameters of this algorithm are first determined. To ensure that the algorithm ran efficiently and without biases owing to divergent iterations ([15], chapter 15), we determined appropriate hyperparameters through initial runs of the algorithm. Our approach requires that we repeatedly call Stan to perform sampling (i.e. each time step 1 is called), and we assumed that the hyperparameters were initially the same across each run, but we allowed Stan to automatically tune these.

Unlike for the Poisson model, we found efficient inference for the unrestrictive model to depend more acutely on the starting points of the Markov chains. To minimize the length of the warm-up period, we initialized all Markov chains at the *maximum a posteriori* (MAP) estimates returned by an initial optimization step (see §2f). The risk of this is that we fail to explore the full posterior space resulting in biased estimates ([15], chapter 12), but we found this risk more than compensated for by the gains it provided for sampling efficiency.

### (f) Optimization

Both models permit rapid estimates of the parameters to be obtained using optimization. These estimates do not have uncertainties associated with them but provide a guide to the consequences of making different assumptions on the resultant estimates. For both models, we replace each of the algorithm’s Gibbs-type sampling steps with algorithms that maximize the conditional log-posterior; our aim is then to produce MAP estimates. This iterative approach may reach local maxima, but, in practice, we have not found this to occur.

For the Poisson model: in step 1, we use an analytical result for the MAP estimate for $R_{t}$; in step 2, our search space is discrete and we just take case count for each onset time which maximizes the posterior; in step 3, we use the default optimization algorithm provided by base R’s *optim* function.

For the unrestricted model: in step 1, we use the same approach to determine estimate cases as for the Poisson model (i.e. its step 2); in step 2, we use R’s *optim* function using the L-BFGS-B optimization algorithm [17].

### (g) Estimation of $R_{t}$ when assuming case data are perfect

In some results, we showcase the difference in $R_{t}$ estimates between our approach, which simultaneously estimates case counts, and a naive approach which treats the case data as being perfect. To do this, we used Stan’s NUTS algorithm [16] to perform estimation since it allowed us to make diverse sets of assumptions about the renewal model and reporting delay processes.

### (h) Prior choice

Throughout this work, we used relatively wide and generally uninformative priors (see table 1).

**Table 1.** Priors used in all analyses. Note that all parameters were assigned independent prior distributions; $\mu_{\theta }$ , $\sigma_{\theta }$ refer to the mean and standard deviation of the reporting delay distributions which are assumed to be parameterised by gamma distributions throughout this work.

parameter	prior	justification
$R_{t}$	gamma (mean = 5, s.d. = 5)	conservative prior giving ∼ 82% weight for $R_{t}>1$
$\mu_{\theta }$	gamma (mean = 10, s.d. = 15)	prior 2.5–97.5% interval: <0.1–53 days
$\sigma_{\theta }$	gamma (mean = 5, s.d. = 15)	prior 2.5–97.5% interval: <0.1–47 days
I(t)	discrete-uniform ( C(t) , $10\;\max_{t,t^{\prime }}\left(C(t|t^{\prime })\right)$ )	empirical prior across wide range

### (i) MCMC convergence monitoring

Throughout this study, we performed diagnostic checks that the MCMC had converged. We diagnosed convergence if $\hat{R}<1.2$ (in the majority of cases, $\hat{R}<1.01$) for all parameters, and we used the *posterior* R package [18] to calculate this statistic.

### (j) Reproducibility and reliability

The methodological work underpinning this article is technical, and we used multiple approaches to ensure that our results are reproducible. We used the *targets* R package to set up a reproducible data analysis pipeline for producing all our figures [19]. We used the *renv* package to manage our R environment [20], which makes it easier for others to duplicate our software environment when rerunning our results.

To allow others to use our methods, all code underlying our inference algorithms was developed into two separate R packages: *incidenceinflation* [21], corresponding to the Poisson model; and *incidenceinflationstan* [22], corresponding to the unrestricted model. Each of these is straightforwardly installable using only a single line of R code.

To mitigate against the risk of coding errors, we wrote unit tests resulting in a high testing coverage for both R packages. We also used continuous integration testing to minimize the risk that changes introduced into the code at later times did not break earlier tests.

The materials to return the analyses in this paper are freely available in a GitHub repository [23].

## 3. Results

We illustrate how our method performs using a combination of simulated and real outbreak data. In §3a–c, we use simulated data. In §3d*,*e, we use real outbreak data for dengue fever and measles, respectively.

### (a) Our approach can simultaneously estimate infections, reporting delays and $R_{t}$ given case histories

We begin by simulating an outbreak using equations (2.1) and (2.2) assuming a COVID-19-like serial interval distribution with a mean of 4.6 days and a standard deviation of 4.8 days [24]. We assume a time-invariant reporting delay distribution characterized by a gamma distribution with a mean of 10 days and a standard deviation of 3 days.

**Figure 2.**
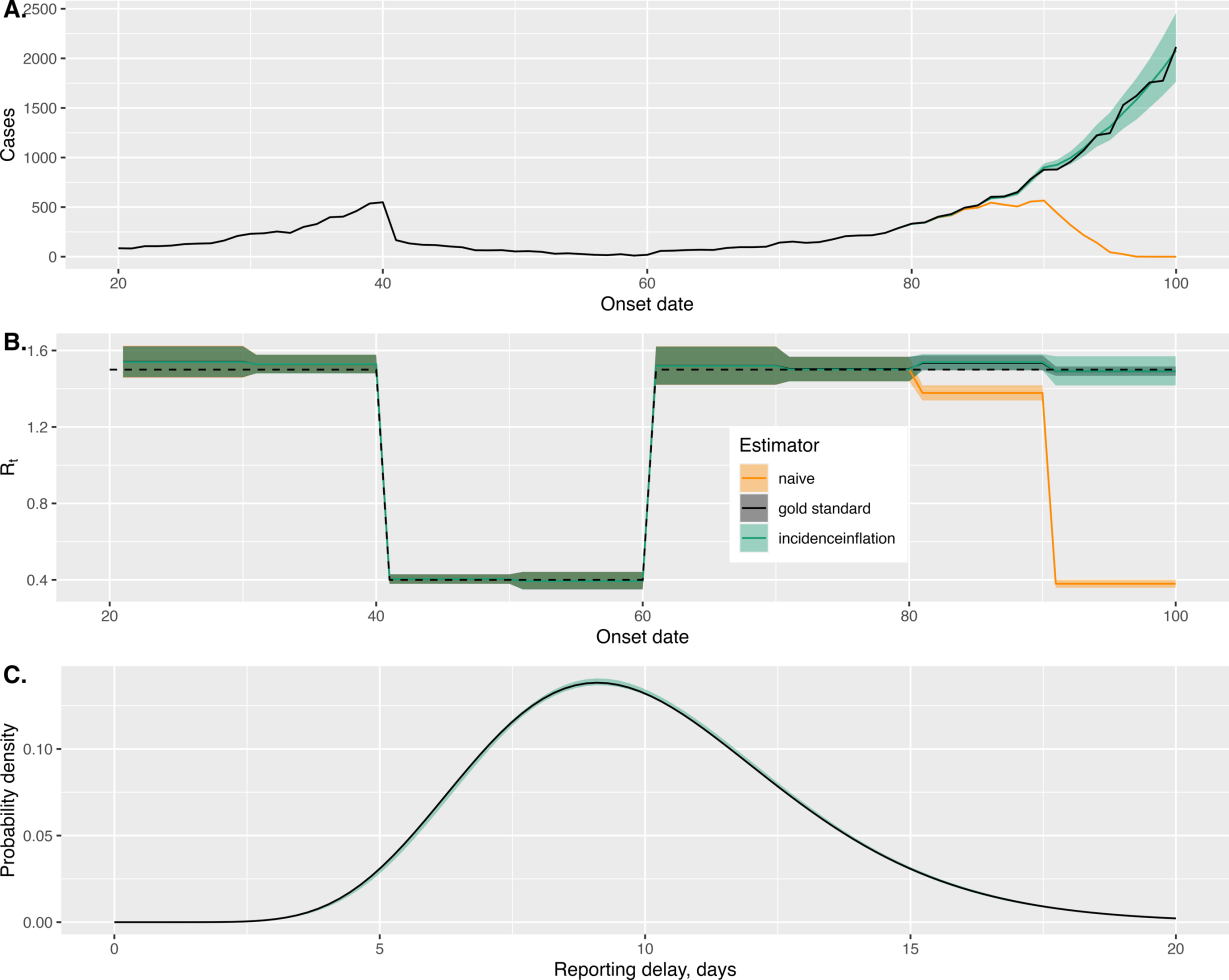
Simultaneously estimating reporting delays, case counts and **$R_{t}$** for a simulated dataset. For this analysis, we consider ourselves at an observation date of 100 days post the start of the outbreak. In (A), we show the observed cases at this observation date (orange line), and the true cases which are eventually reported (black line); our estimates are shown in green (the line represents posterior median). In (B), we show the true $R_{t}$ values (dashed black line); we also show three sets of estimates: those from a naive approach which assumes the case data are perfect; another representing the gold standard where we know the true case counts; and a final set using our method. In (C), we show the true reporting delay distribution (black line) and are recovered estimates (green shading). In all panels, the uncertainties shown represent the 2.5–97.5% posterior intervals.

In figure 2*a*, we show a snapshot of the observed cases at t=100 (orange line) and the true case count (black line). The stark divergence between these two series illustrates the unreliability of recent case observations, which, in our model, underestimate the true case counts. On the same plot, we show our estimates of the true case count (green line shows posterior median estimates; 95% central credible interval estimates shown by shading), which are reasonably close to the ground truth. This also illustrates that the uncertainty in our estimates of cases grows the closer to the present day we consider. This is because, intuitively, we have the least information about those cases occurring most recently.

In figure 2*b*, we show two sets of $R_{t}$ estimates produced using only information available 100 days after the outbreak onset: *naive* estimates assuming a standard Poisson renewal model where the reported cases are assumed perfect; and the estimates of $R_{t}$ from our *incidenceinflation* approach. We compare these with the ground truth values (dashed line). We also compare these with $R_{t}$ estimates under the *gold standard* scenario, where the case counts are perfectly known; this effectively represents the situation where we are looking back from a much later vantage point.

Unsurprisingly, this shows that blind reliance on the observed cases results in downwardly biased $R_{t}$ estimates. It also shows that our approach can generate reasonable $R_{t}$ estimates by leveraging the information inherent in the case histories. The estimates we produce have similar point estimates to the gold standard scenarios, although ours have wider uncertainty intervals for the most recent period owing to the uncertainty about the true case count.

In figure 2*c*, we display the reporting delay distribution (black line) and that recovered by our method (green line and shading), which shows them in good agreement.

**Figure 3.**
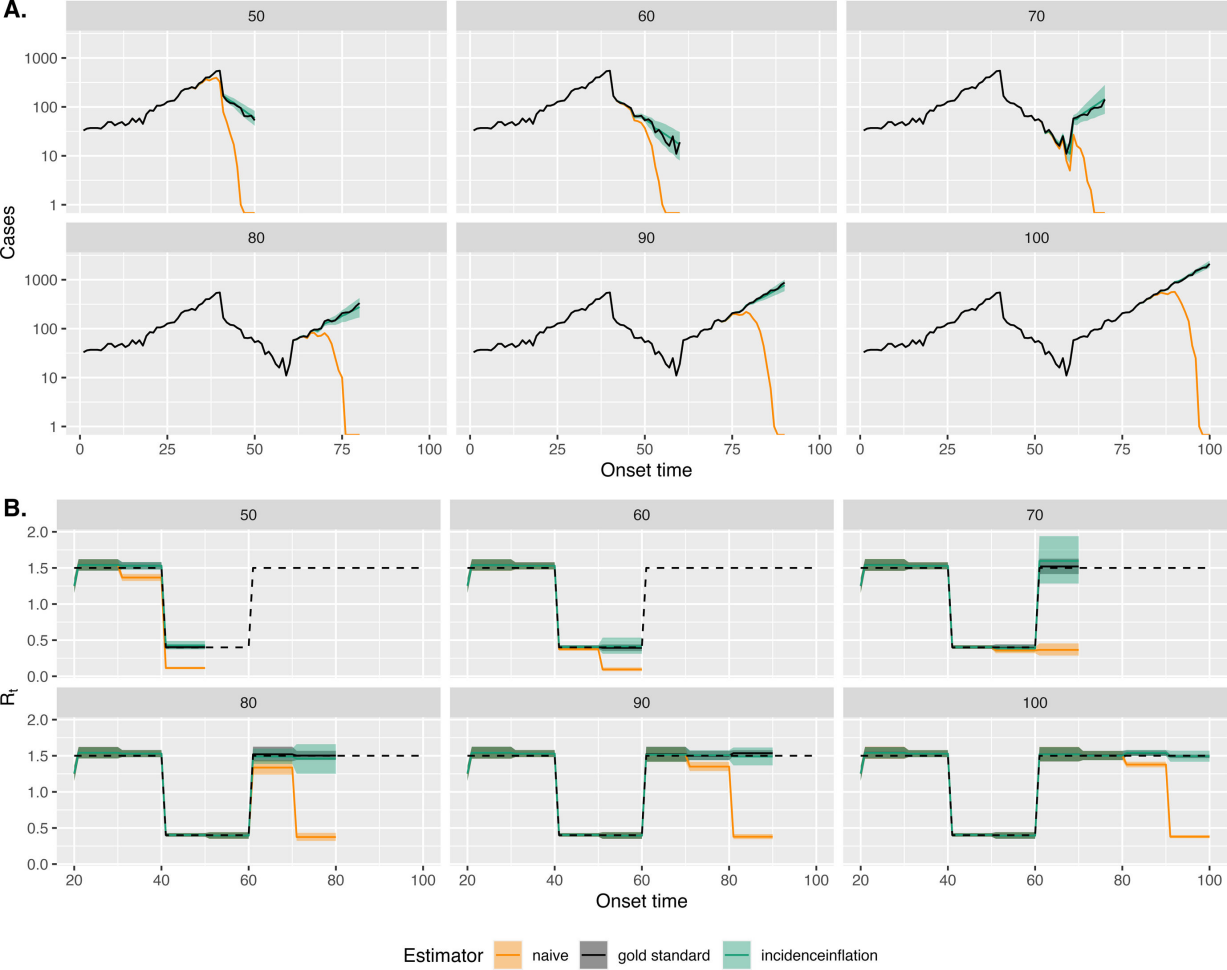
Reliable estimation of case counts and **$R_{t}$** across a range of vantage points. In both (A) and (B), each plot shows estimates assuming an observer at the labelled number of days post the start of the outbreak; e.g. ‘50’ denotes an observer producing estimates at 50 days post the start of the outbreak.

Since we have access to both true and observed cases over time, we repeated the same exercise as above but considering different observation times. In figure 3, we show estimates of the cases (in (*a*)) and $R_{t}$ (in (*b*)) across a range of times throughout the outbreak. Figure 3*a* shows that for each vantage point considered, our uncertainty bounds for case counts contained the actual values. It also illustrates that our method is able to handle epidemics that are growing or shrinking in size.

Figure 3*b* shows that the naive approach which uses only the observed case counts leads to underestimates of $R_{t}$ at each vantage point. It also shows that our approach and the gold standard approach, given perfect information, produce broadly similar estimates of $R_{t}$; albeit our estimates typically have greater uncertainty associated with them, reflecting the additional uncertainty over the case counts.

### (b) Our method can account for reporting delays which vary over the epidemic but estimates of case counts and $R_{t}$ are sensitive to assumptions made about the time periods when the delays are constant

Reporting delays often shorten during outbreaks as health systems improve. We now simulate an outbreak with shortening reporting delays with an Ebola-like serial interval distribution characterized by a mean of 15.3 days and a standard deviation of 9.3 days (the all-countries serial interval reported in [25], table 6). We allow for superspreading by generating the outbreak assuming a negative binomial renewal model with κ=5. We also use a negative binomial model to fit the data.

**Figure 4.**
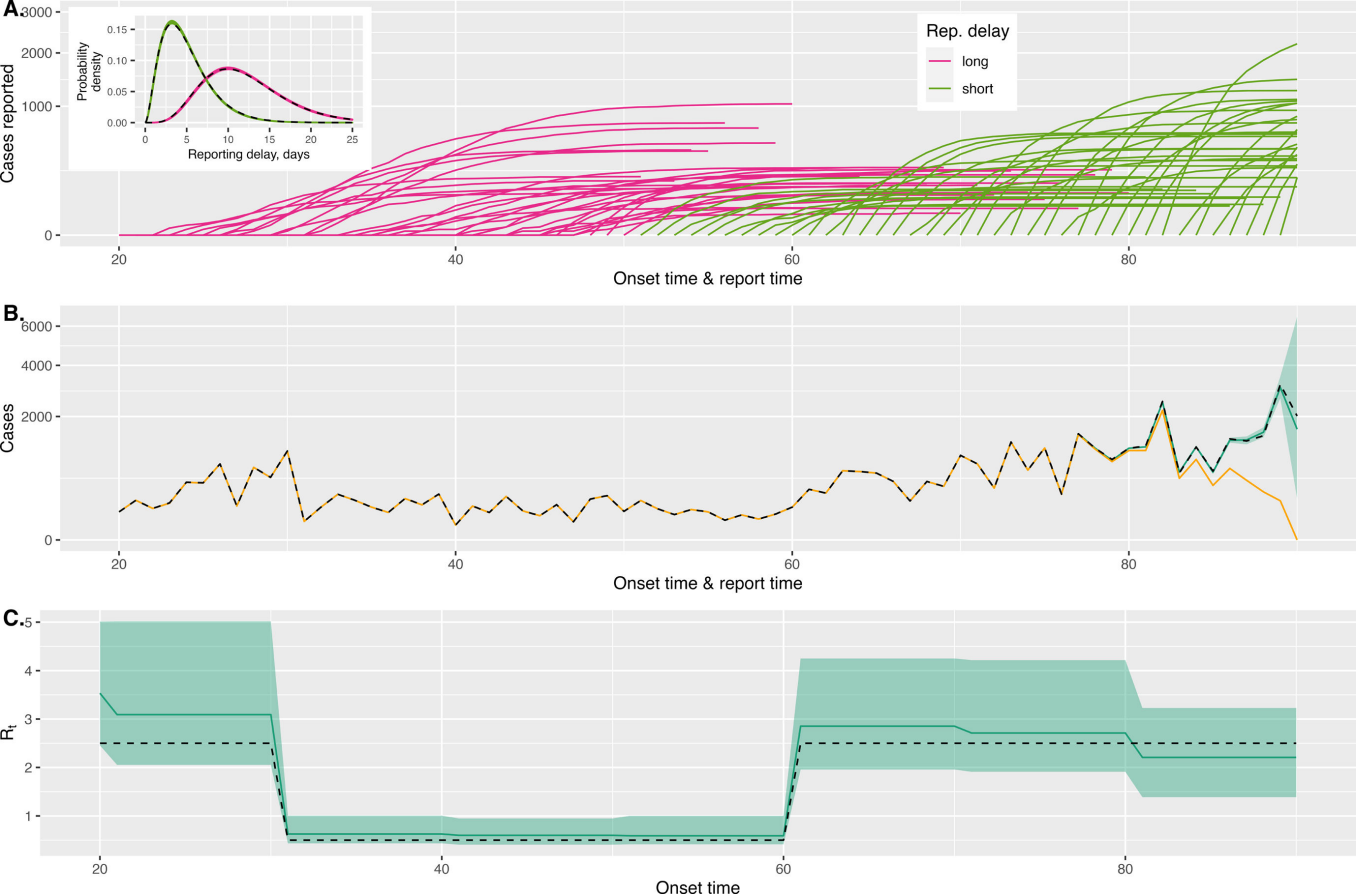
An Ebola-like outbreak with shortening reporting delays. (A) Shows the trajectories of retrospectively reported cases with onset on a particular day (where each line first stems from the horizontal axis). Here, we colour the lines according to whether they occur before or after a change in the delay period at an onset time of 50 days; before this point, the reporting delay distribution has a mean of 12 days and a standard deviation of 5 days; after it, the mean is 5 days and the standard deviation is 3 days. In both time periods, the reporting delay distribution is characterized by a gamma distribution. The inset figure in (A) shows the true reporting delay distributions (dashed black lines) and posterior draws of the densities (coloured lines). In (B), we show the true cases with onset on each day (black dashed line); we consider a vantage point of 90 days after the outbreak began and show the currently recorded time series case counts (orange line). The green line shows our method's posterior median estimate of the case count and the ribbon shows the 2.5–97.5% posterior distribution estimates. In (C), we show the true $R_{t}$ values used to generate the outbreak (dashed lines) and the values recovered by our method (green lines show posterior medians and the uncertainty ribbon shows the 2.5–97.5% posterior distribution estimates).

In figure 4*a*, we show how the trajectories of retrospectively reported cases change throughout the outbreak: the reporting delays are initially long and shorten after the first 50 days of the outbreak, and this is characterized by more slowly rising trajectories and then more abruptly increasing trajectories.

We allow our model to estimate different reporting delay distributions in the first 50 and remaining days of the outbreak; in the inset panel in figure 4*a*, we show that our model can accurately estimate each of these distributions.

In figure 4*b*, we show the true case series (dashed black line) and the reported cases (orange line) from an observer’s point of view at 90 days since the outbreak began. This shows that the case counts nearest the current time are most underestimated. Our method explicitly accounts for these reporting delays and so can accurately estimate the true case counts (green line with uncertainty shown by shading). The result of this is that the $R_{t}$ estimates for the most recent time period remain reasonable (figure 4*c*).

In a real outbreak, of course, it is unknown when and if changes to the reporting delay distribution occur; this means that the results we show in figure 4 represent a best-case scenario. We now consider a more realistic situation when we do not know if and when the reporting delays change. We produce estimates of the various epidemiological quantities for the same Ebola-like outbreak under three scenarios: one matching the best-case scenario already described; another where we assume there are no changes in the reporting delays; and a final situation, where we assume that the reporting delay distributions remain the same within bins of width 30 days (shorter bins caused issues with estimation owing to insufficient data to produce reliable estimates). We also use this as an opportunity to show that our method allows for rapid estimates using optimization rather than sampling methods; these estimates do not have uncertainty associated with them but provide a quick way to get an idea of the implications of different assumptions on the resultant estimates. In our experience, these first optimization steps can be very useful as part of a full Bayesian workflow.

In electronic supplementary material, fig. S1, we show the estimates of the reporting delay distribution in each of these three circumstances. This shows that in all circumstances, fewer than 10 iterations (taking only a few minutes for each situation) of the optimization algorithm were sufficient to converge to reliable estimates. In those situations where the reporting bins overlapped only a regime where only a single reporting delay was present, our estimates of the reporting delay distribution coverage were close to the actual values. In those situations when the assumed bins overlapped multiple reporting delay regimes, it was a mix of the corresponding reporting delay distributions.

A consequence of mistakenly assuming that reporting delays are constant when, in fact, they change, is biased estimates of the history of cases. In electronic supplementary material, fig. S2A–C, we show how our estimates of the true case counts progress as our optimization algorithm proceeds for each of the three differing assumptions being made about the periods when the reporting delays are constant. In panels *b* and *c*, the reporting delay periods are correctly assumed constant in the last of the subperiods, and the estimated case counts are close to the actual values. In panel *a*, corresponding to the situation when we fail to account for any change in reporting period over the outbreak, we underestimate the most recent reporting delays (see electronic supplementary material, fig. S1C) meaning that we generally overestimate the most recent case counts.

A consequence of overestimating cases is that we overestimate $R_{t}$, although the degree of overestimation here is not substantial (electronic supplementary material, fig. S2D). Whereas the estimates of $R_{t}$ when we correctly characterize the reporting delay distributions are slightly below the true values (electronic supplementary material, figs. S2E and F). This figure also shows how information between estimates of case counts and $R_{t}$ is shared throughout the course of the algorithm: as case counts fall, so does $R_{t}$ and vice versa.

### (c) Uncertainty in recent case data results in conservative estimates of the probability that an outbreak goes extinct

We now simulate a waning Ebola epidemic assuming reporting delays are described by a gamma distribution with a mean of 10 days and a standard deviation of 3 days. We simulate it such that no cases are reported to have occurred in the last week and consider determining the probability that the epidemic has finished. We compare three approaches; all of these scenarios are based on the same underlying epidemic but suppose differing access to information or use different approaches to calculate this probability: one which has perfect case and $R_{t}$ information as a gold standard; another which naively uses the reported case counts but has perfect $R_{t}$ information; and our approach which uses information on reporting delays to inflate the case counts and accounts for uncertainty in both case counts and $R_{t}$.

**Figure 5.**
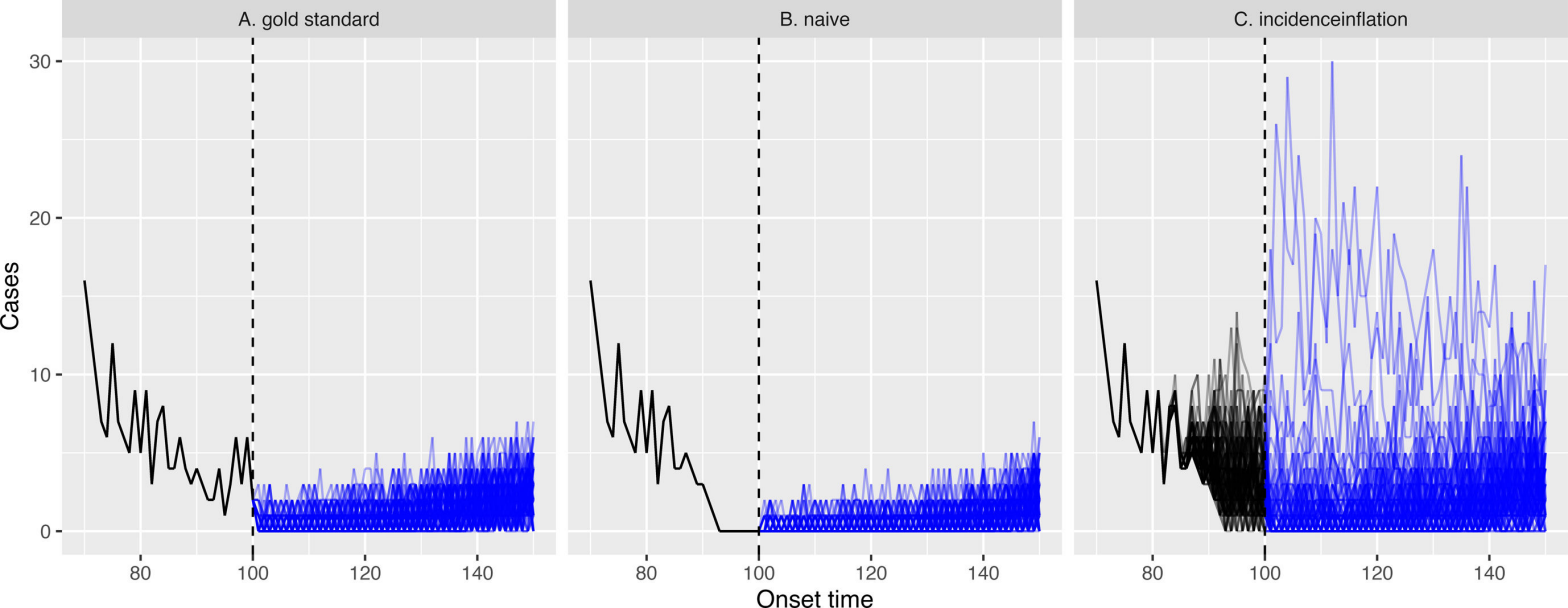
Outbreak projections for a waning epidemic for three different scenarios. In each panel, we consider an observation time of 100 days after the onset of the outbreak; the black series represent the past case series: for each of panels (A) and (B), these are a single series; for panel (C), it represents a family of series since our method nowcasts cases; the blue lines show projections assuming transmission remains the same in the future. For the projections, 100 iterations were used.

In figure 5*a*, we show the projections for the gold standard scenario. In this scenario, the actual case count before an onset time of 100 days is perfectly known, and this approach produces daily case projections that are all lower than 10 for the next 50 days. In figure 5*b*, we show the naive scenario projections: these are similar but the upper bound of the projections are lower than the gold standard scenario. In figure 5*c*, we show the projections from our method which reconstructs past cases and includes this uncertainty (and uncertainty in $R_{t}$) when making projections. Owing to these additional uncertainties, there is considerably more variability in the projections.

We quantify the probability that an outbreak is over by performing 1000 projections for each scenario and counting the fraction of them with zero cases 50 days in the future. For the gold standard approach, 71% of projections resulted in the epidemic dying out. The naive approach results in overly confident determinations that the epidemic has died out, with 86% of projections resulting in it dying out. The corresponding probability for our method was 67% owing to the added uncertainties incorporated into our approach. Our method’s estimates are therefore conservative and risk-averse, which is appropriate for policymaking.

### (d) Dengue fever in Puerto Rico

We now consider a time series of laboratory-confirmed dengue fever cases from Puerto Rico collected by the Puerto Rico Department of Health and Centers for Disease Control and Prevention; this dataset was originally analysed by [26] who developed a statistical model for predicting dengue cases accounting for the delay between the time of case onset according to a clinician and the time when the laboratory results were returned.

The data are weekly, and with such data comes the risk that cases occurring earlier in each week could spawn cases that were reported later in the week. We consider this a minor concern since the serial interval for dengue is thought to be well in excess of a week even at high temperatures where it is shortest [27,28], accounting for both the human and mosquito stages of the transmission cycle. Here, for simplicity, we assume a temperature-independent serial interval of four weeks which is the median estimate reported by [28] at 25°C; to allow a wide range of serial intervals potentially encompassing some fluctuation owing to temperature variation, we assume a standard deviation of two weeks.

We first assessed whether the reporting delay period was consistent throughout the analysis period by considering the empirical CDFs for each onset date within each subperiod. This showed there was minimal variation in the reporting day distribution over time (electronic supplementary material, fig. S3), and we assumed a fixed delay distribution throughout our period of analysis in our modelling.

**Figure 6.**
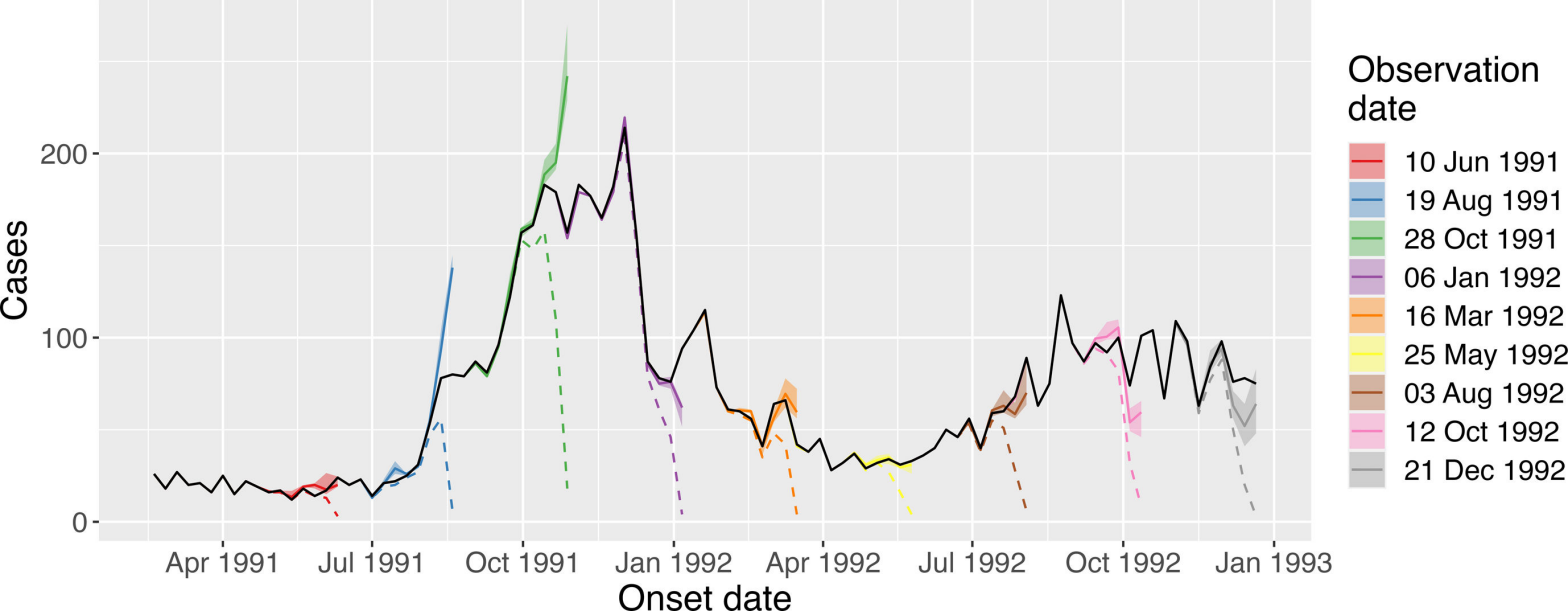
Dengue case estimation across a range of observation dates. The black line shows the true case count (i.e. the reported case counts at a time long after the corresponding onset date). We consider eight observation dates, where we assume there is access only to the cases reported up until that date (dashed coloured lines). For each of these observation dates, we used our method to infer the true case counts, and the posterior median estimates are shown by coloured lines with 2.5–97.5% posterior quantiles shown by uncertainty ribbons.

We fitted our model to the dengue case data and used it to estimate the true case counts at a range of observation points. Since we had access to the numbers of cases that were eventually reported for each onset date, we were able to assess the performance of our model at recovering the true case counts. In figure 6, we show the true case counts (black line), the reported cases corresponding to eight different observation dates (dashed lines) and the case counts recovered by our method (coloured solid lines and uncertainty intervals). Our method was generally good at recovering the true cases the further they were back in time from the observation date. The estimates of case counts were more variable the closer these were to the observation date, although these always represented a substantial improvement over using the raw reported case counts. Generally, our case estimates were best for those periods of relative stasis in the case counts; in those periods where there was epidemic growth, our estimates overshot their true counts, probably owing to changes in the reproduction number over short periods.

We next focused on $R_{t}$ estimation from these case data, and we considered the same eight observation dates. In electronic supplementary material, fig. S4, we show three sets of estimates: naive estimates which assume the true case counts were given by the cases reported up until each observation date (i.e. the dashed orange lines in figure 6); the gold standard estimates, which had access to the true case counts for each date—these assume perfect foresight over the case counts; and a set of estimates from our method. For each observation date, the naive approach substantially underestimated $R_{t}$ for the most recent period. Our set of estimates were always higher than those produced by the naive approach, and the uncertainty from our method was generally higher than from the other two methods, owing to the additional uncertainty from not knowing the true case counts. Generally, the $R_{t}$ estimates output by our method were closer to the gold standard estimates when the estimated case counts were closer to the true counts: for example, for an observation date of 10 June 1991, the estimated case counts were similar to the true values (figure 6; red), and the $R_{t}$ estimates between our method and the gold standard were similar; on the 19 August 1991, our case estimates overshoot the true case counts (figure 6; blue), and our $R_{t}$ estimates also overshoot.

### (e) 2013–2014 measles outbreak in the Netherlands

We now explore the performance of our approach using daily data from a measles outbreak in the Netherlands in 2013−2014 [4]. This dataset, unlike most case count series, contains information on both the onset time of the case and the time the case was recorded. We assume a serial interval distribution parameterized by a gamma distribution with a mean of 11.7 days and standard deviation of 2.15 days (the mean is the overall estimate reported by [29]; the standard deviation is the mean across all standard deviations reported in their table 3).

We tried fitting the data with both Poisson and the negative binomial renewal models; the fits were similar, while the Poisson model estimates were much faster to generate. So we used the Poisson model to generate the raft of results we present.

**Figure 7.**
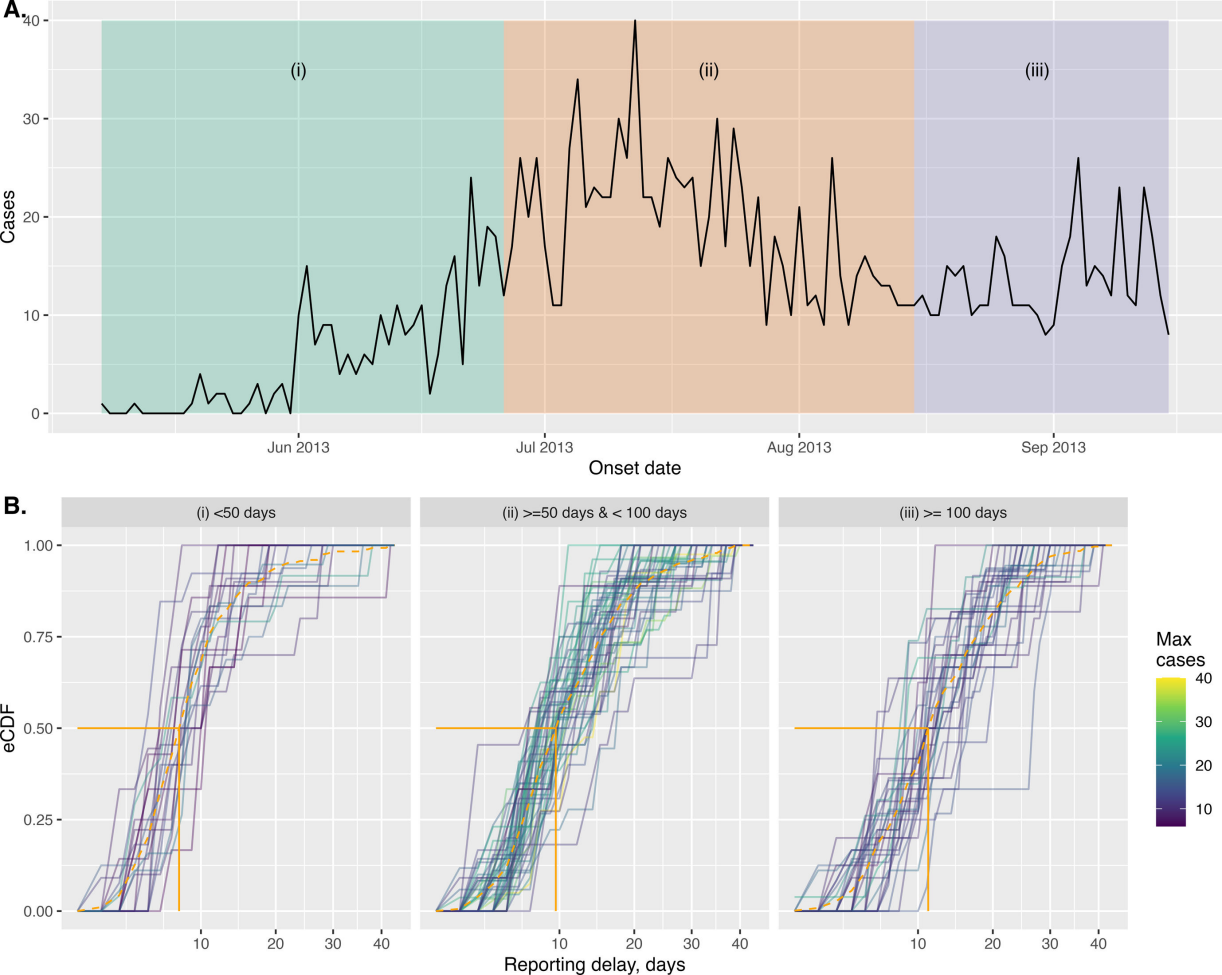
Netherlands measles outbreak and reporting delays. (A) Shows the daily measles case counts and labels three subperiods. (B) Visualises the reporting delay distributions in each of the corresponding subperiods; in each panel, the dashed orange line shows the mean empirical cumulative distribution function (eCDF) and the solid orange line indicates the median reporting delays. Each (non-orange) solid line indicates an eCDF corresponding to a particular onset date within that period. The (non-orange) lines are coloured according to the maximum cases observed for each of the onset dates.

We constructed empirical CDFs for the reported cases, assuming the last reported case counts represented the true counts for each onset date for three distinct periods during the outbreak (figure 7). This showed that the delay periods generally increased throughout the outbreak; we allowed for this during inference by allowing separate estimates of the reporting delay distribution in three distinct periods (those shown in figure 7*a*).

**Figure 8.**
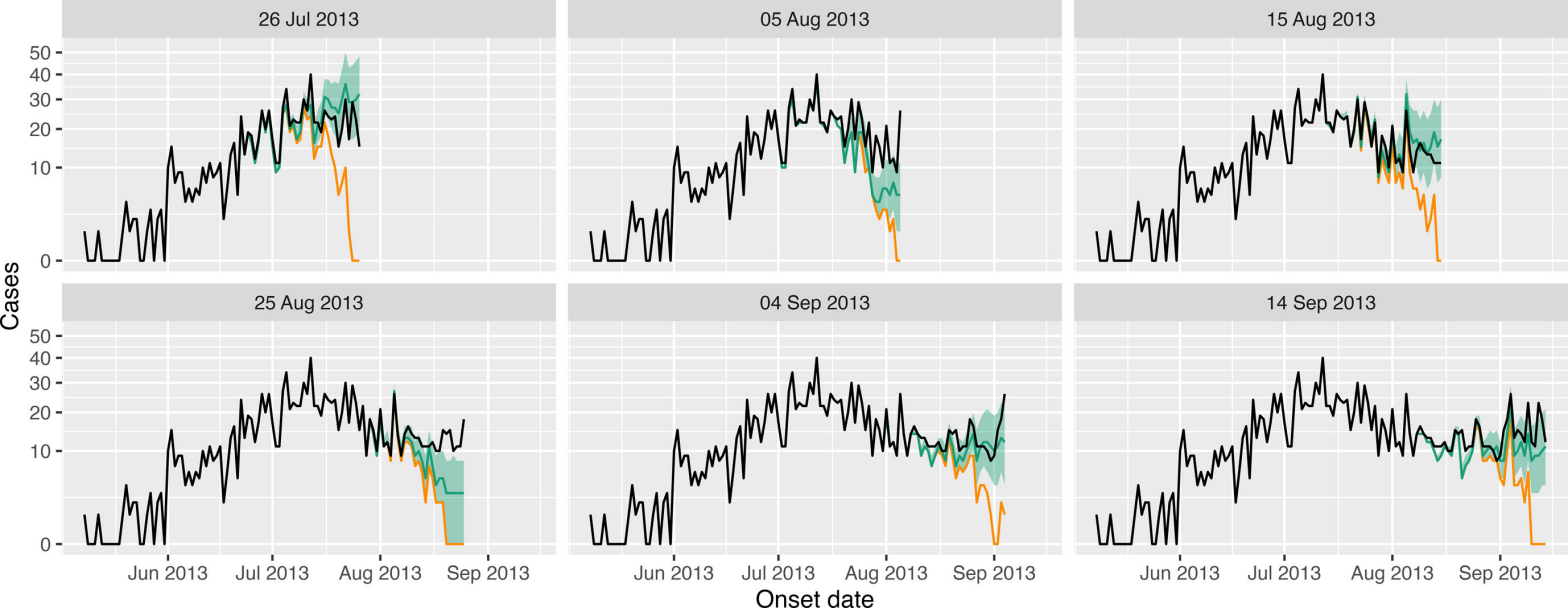
Measles case estimation across a range of observation times. Each panel shows the cases reported up until a specific observation time (in orange); the black lines show the true case counts; the green lines show our estimates of the case counts (posterior medians) and the uncertainty ribbons show the 2.5–97.5% posterior intervals.

In figure 8, we show the true and recovered case counts using our method across six different observation dates; we also show the cases reported up until that observation date. Our estimates were generally comparable with the true case counts with the exception of 25 August 2013, when our estimates undershot the true counts.

**Figure 9.**
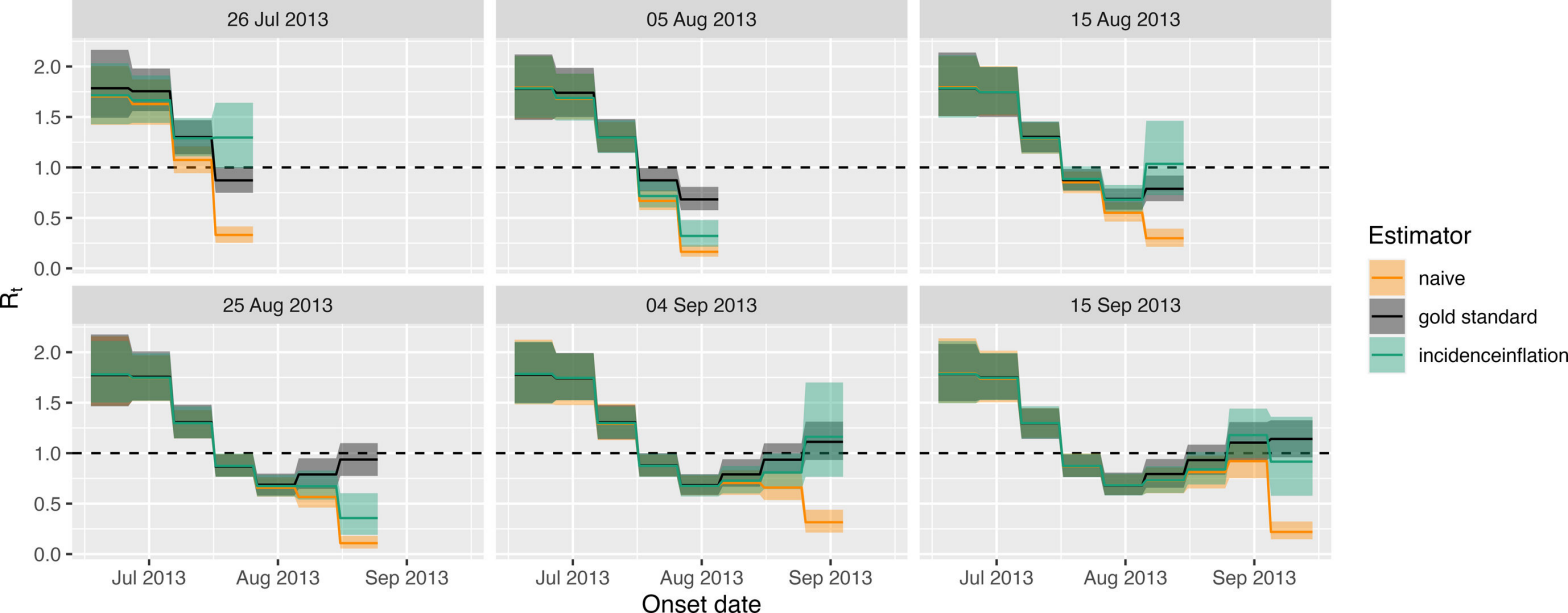
Measles **$R_{t}$** estimation across a range of observation times. Each panel corresponds to a particular observation date. Within each panel, we present three sets of $R_{t}$ estimates: *naive* estimates where we assume the reported cases are perfect; gold standard estimates, where we have access to the true case counts; and a set produced by our method, *incidence inflation*. The uncertainty ribbons show the 2.5–97.5% posterior estimates from each method; the lines show the posterior median estimates.

In figure 9, we show the corresponding $R_{t}$ estimates for each of the six observation dates. We also show naive estimates corresponding to assuming the cases reported up until the observation date are correct, and gold standard estimates, which assume access to the true case counts. Across the six scenarios, our estimates always improved upon the naive estimates. The uncertainty bounds from our methods, however, did not always contain the true estimates.

Overall, these suggest that our method does not allow sufficient uncertainty in either the estimated case counts or $R_{t}$ values. This suggests model misspecification. The cases estimated by changing a Poisson model to a negative binomial model were not greatly changed (see electronic supplementary material, fig. S5 for one example). This suggests that the problem may lie with the reporting delay model, and electronic supplementary material, fig. S6 suggests that this may be owing to bunching together of reported cases. A beta-binomial reporting model implicitly allows the probability of reporting to fluctuate but the fits of the model to the data remained relatively poor (electronic supplementary material, fig. S7), and we discuss in §4 potential future research to handle such data.

## 4. Discussion

Outbreak data are imperfect and one of the key corruptions of these is from reporting delays, which means that reliance on observed cases alone produces downwardly biased estimates of $R_{t}$. In real outbreak scenarios, analysts know that recent case data are not reliable and analyses are then restricted to a so-called trusted region which fails to produce contemporaneous $R_{t}$ estimates. Here, we present a framework that explicitly acknowledges reporting delays and makes use of data representing a shifting history of case series. Our method is Bayesian and jointly estimates the true cases, the reporting period and $R_{t}$, accounting for uncertainty in each of these sources. Our method allows up-to-current estimates of $R_{t}$ providing policymakers with timely information about which to act.

Our method is only as good as the assumptions we make about the underlying disease transmission process and the reporting delays. Here, we assumed that $R_{t}$ was piecewise-constant, and these pieces must be decided upon *a priori*. For the dengue data we analysed, it looks as if there were often shifts in $R_{t}$ within the pieces assumed, which sometimes caused our estimates of cases to overshoot the mark (figure 6). Of course, we could choose to make these windows narrower, but the process of selecting appropriate piece widths could be automated using Bayesian non-parametric processes [13]; alternatively, adapting our method to estimate $R_{t}$ in a more continuous fashion, for example, by adapting EpiEstim [30] and EpiFilter [31] could be worthwhile. Similarly, we assume that the distributions characterizing reporting delays remain constant within given time intervals, and this must be decided upon before running our inference approach—this process can, however, be informed by visual inspections of the data such as those we show here (electronic supplementary material, fig. S3).

Throughout, we assumed that there was a binomial observation process which governed the numbers of cases observed in a fixed interval equation (2.2). We demonstrated that, for the measles data in particular, this was not a good approximation to the process governing how cases are reported, where they often appear to occur in groups. This is possibly because finding one additional case may lead to a whole group of their infected contacts also being located, violating an implicit assumption of the binomial distribution, and our estimates lacked uncertainty owing to this. We tried a beta-binomial distribution here but this did not greatly improve the fit while adding substantially to the runtime. Further work building a more mechanistic model of how reporting delays arise should lead to more appropriate estimates of uncertainty. In addition, incorporating the flexibility of more heuristic approaches to case count estimation (for examples, using the methods of [5]) could allow a more dynamic and accurate depiction of reporting delays.

The predominant approaches for $R_{t}$ estimation, like EpiEstim [30], are inherently backward looking. This means that estimates of $R_{t}$ are probably inefficient since they use case data only up until that time point. A better approach would be to use all the information in the case data; methods such as EpiFilter do this [31]. Assuming piecewise-constant $R_{t}$ values is a sort of halfway house between each of these two extremes since assuming that $R_{t}$ is fixed forces case data later than a time point to be used to determine estimates. But our estimates ignore all information after the end of a piece, and future work could consider how to incorporate this information. In addition, there is merit in trying to extend our method to be more natively able to handle more continuous prior distributions for $R_{t}$. Our unrestricted model can handle this but at quite a cost to computational runtime. Efficiency savings by using different sampling strategies may be possible, and particle filtering approaches may be fruitful to consider.

Computational methods for estimating key epidemiological quantities such as $R_{t}$ either implicitly or explicitly make assumptions about the quality of the underlying data on which these are based. Predominant approaches to $R_{t}$ estimation implicitly assume the case series are perfect, which ignores the manifold sources of errors in these. Here, we considered one predominant source of measurement imperfections for disease cases—that owing to delays in the time taken for cases to enter health systems—but there are many other types of measurement errors. One such measurement error comes from underreporting, and cure models may prove useful in extending our framework to account for this [32]; another form is idiosyncratic errors where cases may be either above or below their true counts. Indeed, the measles data we considered here probably had a more complex form of reporting delay than that embedded in our model, and further work is needed to develop better models for these. More generally, the predominant statistical approaches to $R_{t}$ estimation make arbitrary assumptions about the nature of the noise within the renewal approaches. Future work to produce models that more mechanistically account for how this noise arises should lead to estimates with more appropriate uncertainties.

## Data Availability

All data underlying this study are available in our public GitHub repository [23]. Supplementary material is available online [33].

## Footnotes

^1^

https://github.com/ben18785/incidenceinflation
